# Radiological Control of the Floating Mass Transducer Attached to the Round Window

**DOI:** 10.1155/2013/902945

**Published:** 2013-11-12

**Authors:** Ingo Todt, G. Rademacher, J. Wagner, P. Mittmann, Dietmar Basta, Arne Ernst

**Affiliations:** ^1^Department of Otolaryngology at ukb, Hospital of the University of Berlin, Charité Medical School, Warener Street 7, 12683 Berlin, Germany; ^2^Department of Radiology at ukb, Hospital of the University of Berlin, Charité Medical School, 12683 Berlin, Germany

## Abstract

The surgical rehabilitation of mixed hearing losses can be performed by coupling the floating mass transducer of the Vibrant Soundbridge to the round window. The quality of coupling the floating mass transducer to the round window is crucial for the audiological outcome. It was the aim of this study to further observe the different patterns of floating mass transducer position at the round window. We compared twenty patients with mixed hearing loss implanted with a floating mass transducer attached to the round window and 24 surgeries between 5/2007 and 6/2010. An evaluation of the chronological observation of the flat panel angiography-controlled position of the floating mass transducer at the round window with relation to the surgical report and the audiological outcome was done. We observed no changes in the mean pre- and postbone conduction thresholds. The floating mass transducer position was variable and could be radiologically classified and correlated with the audiologically outcome. A learning curve was observed from the earlier to later implantations. Postoperative, radiological evaluation of the location and angle of the floating mass transducer by means of flat panel tomography allowed us to classify the floating mass transducer position at the round window into 4 groups.

## 1. Introduction

The Vibrant Soundbridge middle ear implant (VSB) was introduced in the late 90s for the treatment of high-frequency, purely sensorineural hearing loss (SNHL) as well as for patients with SNHL who were unable to use conventional hearing aids due to problems such as chronic otitis externa. Colletti et al. [1] were the first to attach the floating mass transducer (FMT) to the round window, extending the indication criteria of the VSB to include patients with mixed hearing loss. Subsequently, different coupling methods for the FMT arose (e.g., at the stapes, oval window, or third window, as well as combined with TORPs and PORPs), which allowed the surgeon to choose a flexible, individualized treatment option while performing the procedure in the surgical theatre. 

Various centers have described their experiences with attaching the FMT at the RW [2–5]. Temporal bone studies using functional laser doppler vibrometry (LDV) measurements outlined the importance of the position of the FMT in regard to transfer function when attaching it at the round window [6–9]. Clinically, it is well known that variations in the audiological outcome exist with the round window coupling mode, ranging from unsuccessful attachment to closure of the air-bone gap all the way to excellent functional gain which exceeds the bone conduction threshold.

Postoperative, radiological evaluation of the position of otological implants is a useful tool for quality control, for example, in cochlear implantation [10] and stapes surgery [11, 12]. It is important for the clinical outcome, for making decisions about surgical revisions, and for optimizing the surgical technique.

It was therefore the aim of the present study

- to define the different patterns of FMT position at the RW,
- to correlate these findings with the functional outcome,
- to compare radiological and functional outcome data with the chronology of implantation and the different surgical techniques applied.

## 2. Materials and Methods

### 2.1. Patients

Between May 2007 and June 2010, 24 round window attachments of the FMT were performed in 20 patients and included in this study. All patients had a previous history of multiple middle ear operations. The surgeries were performed by a transcanal approach (surgeries no. 3, 6, 7, 8, 9, 10, 12, 16, 17, 19, 22, and 24) or by a two step surgery in canal wall down patients (surgeries no. 1, 2, 4, 5, 11, 13, 14, 15, 18, 20, 21, and 23). The first step was a decrease of the cavity size by a flap followed by a second step after 3–6 month with the implantation of the FMT.

### 2.2. Surgeries

 In the retrospective part of the study, 7 implantations using different attachment techniques (between May 2007 and May 2009) were performed (see Section 3 for details). All audiological data is based on what was estimated at the first fitting of the audio processor. Radiological scanning by flat-panel angiography was performed after May 2009. 

In the prospective portion of the study, 17 operations (between May 2009 and February 2010) were included using a standardized surgical approach. This approach consisted of the following steps.

- The RW was visualized by removing the promontory lip.
- Low-speed drilling away from the promontory lip was performed at 1000 r/sec.
- Ivalon (a PVA sponge) was placed between the FMT and the round window.
- The FMT was stabilized at the distal end with cartilage and covered with fascia.
- This construction was covered with fibrin glue.

Because they were fitted with the new Amade audio processor (AP), patients number 20 to 24 were only evaluated radiologically.

### 2.3. Radiological Examination

Determination of the FMT-RW position was performed with an Allura Xper FD20 system (Philips Medical Systems, Best, Netherlands), using a flat panel detector. The system's parameters were as follows: entrance field of 22 cm, 274 mAs, 95 kV, 180° rotation, 241 projections, and filter 0.90 mm Cu + 1.00 mm Al and postero-anterior (p.a.). The focus panel distance was constant during the whole rotation at a frequency of 30 pic/s. The 3D angiography was performed in the unsubtracted mode. From this volume data set, the temporal bones were secondarily enlarged (FoV of 100 mm), digitally stored, and sent to an external workstation (Extended Brilliance Workspace, Philips, Cleveland, USA) for the 2D- and 3D-reconstruction. The actual classification of the single scans was performed independently by two ENT surgeons and one radiologist. 

Since a correlation between the radiologic classification and the functional gain could not be observed (Figure 9), a secondary measurement was performed. Additional factors were introduced (AF, APR), which might represent the amount of gain reserve of the system after reaching the obtained functional gain. The assumption was that the more effort the system has to make to reach that FG for an audiological sufficient threshold (in case of insufficient coupling of the FMT to the RW membrane), the less APR remains, and therefore a smaller AF in comparison to a good coupling of the FMT persists.

### 2.4. Calculation of the “Audio Processor Factor” (AF)

 The AF should serve as an indicator for the quality of RW coupling. Two major determinants were considered, that is, the functional gain (calculated as warble tone threshold at the patients preferred volume settings minus postoperative bone conduction threshold) and the so-called audio processor reserve. For a better visualization of the functional gain, the value is multiplied by −1. The audio processor reserve was measured after the fitting by using an audio processor Type 404 (AP 404) in Kuppler mode (with a 2 ccm chamber at 65 dB). The Kuppler value in dB recorded to reach the aided threshold at 2 kHz was subtracted from the Kuppler value to reach the maximum possible AP gain. This value was called AP reserve (APR). The AF was calculated by adding this APR value to the functional gain at 2 kHz. The measurements and calculations were done at and referred to 2 kHz to eliminate a bias induced by a deprivation-related hypersensitivity to higher frequencies [13]. All presented AF estimations were based on the measurements at the first fitting. An exemplary estimation for surgeries 5 and 14 is presented in Figure 8.

## 3. Results

### 3.1. Comparison of the Pre- and Postoperative BC Thresholds

**Figure 1:**
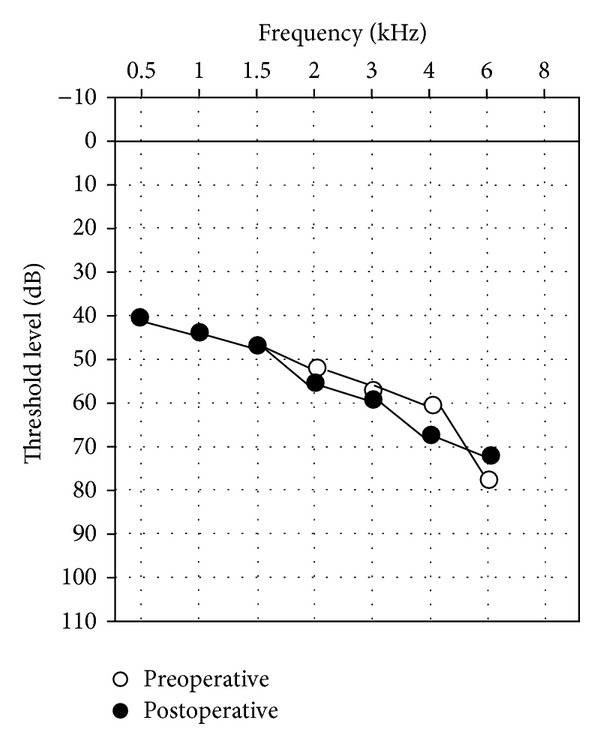
Comparison of the pre- and postoperative BC.

No significant difference (Freq: 0.5; 1; 2; 4; 49.0 dB +/− 14.4 versus 51.5 dB +/− 16.9) was found between the pre- and postop BC thresholds (Figure 1). 

### 3.2. Functional Gain (Overclosure)

A mean functional gain of 10.4 dB with a maximum value at 1,5 kHz, 2 kHz, and 3 kHz of 35 dB and a minimum value at 500 Hz of −25 dB was observed. The individual mean functional gain (.5 Hz, 1 kHz, 2 kHz, 4 kHz/4) is presented (Figure 9).

### 3.3. Radiological Classification

**Figure 2:**
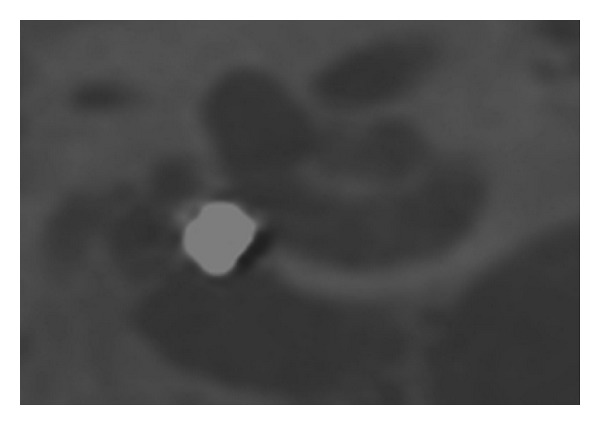
Type IV coupling—FMT directly at the RW in a rectangular position.

**Figure 3:**
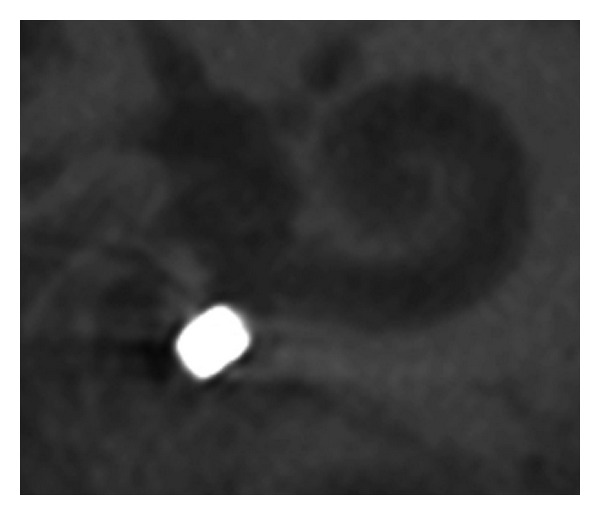
Type III coupling—FMT in contact to RW, not rectangular.

**Figure 4:**
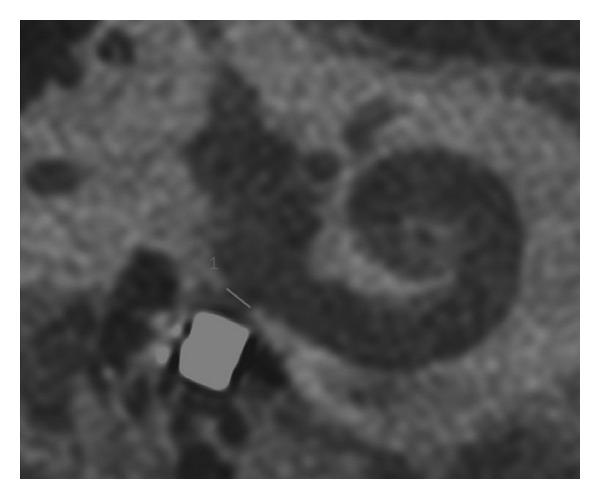
Type II coupling—FMT in RW niche, no direct contact.

**Figure 5:**
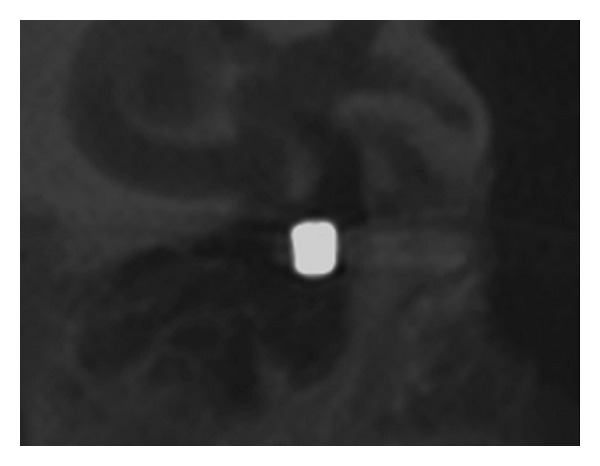
Type I coupling—FMT not in RW niche.

The FMT position in the round window niche as seen on the flat panel angiography could be categorized into 4 different patterns.  Figure 2 shows a so-called *“type 4” pattern* with the FMT lying directly against the round window in a rectangular, length-wise fashion.  Figure 3 shows a so-called *“type 3” pattern* with the FMT not positioned rectangularly, with only partial contact to the round window.  Figure 4 shows a so-called *“type 2” pattern* with the FMT located in the RW niche, but without direct contact.  Figure 5 shows a so-called *“type 1” pattern* with the FMT outside the RW niche. The classification of the FMT position and for the single surgeries was highly reproducible by independent observers (Fleiss-Kappa score: 0.841).

### 3.4. Temporal Changes of the FMT Position with respect to the Surgical Chronology and the Radiographs

**Figure 6:**
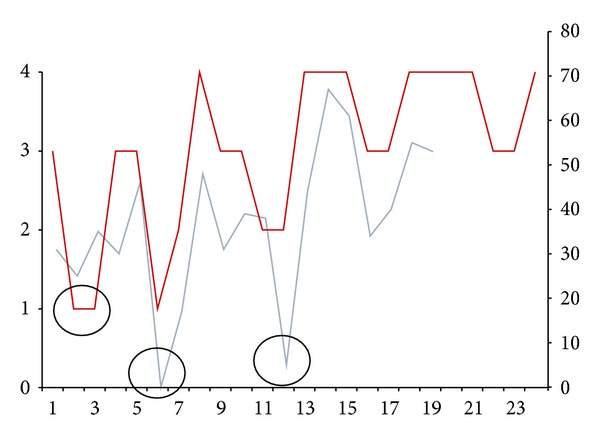
Chronology of implantations. Relationship between radiologic classification (red line) and AF values (blue line). Circles indicate cases of bad coupling. The *y*-axis describes the radiologic classification Type IV to Type I and AF values in dB. The *x*-axis describes the chronology of surgeries 1 to 24.

The first retrospective series (surgeries 1–7) showed the occurrence of a learning curve in terms of optimizing the FMT position within the RW niche (Figure 6), that is, it usually changed from a type 1/2 to a type 3/4 position.

The temporal changes of the AF at the first fitting showed an increase with the number of surgeries over time (Figure 6). 

 Except for patients 2 and 3, the AF and the radiological FMT position (according to the classification) were directly related. In cases 2 and 3, the AF decreased after the first fitting (Figure 6). However, audiologically the air-bone gap could be closed in these two patients as well as in cases 6 and 12. 

### 3.5. Surgical Techniques

We considered different aspects which corresponded to the various ways in which the FMT was positioned. 

In some of our patients, the promontory lip was not or not completely removed and the FMT was merely pushed into the RW niche. This led to a worse radiological position than in the patients where a complete visualization of the RW was possible. This mainly involved the patients in the retrospective group (subjects 2, 3, and 6), but it also occurred in the prospective part of the study (patients 11, 12).

The stabilization of the FMT in the RW niche was performed in different ways. In patients 4, 5, and 8–24, the FMT was supported at the distal end with cartilage, covered with fascia, and stabilized with fibrin glue. Case 7 differs since the cartilage was placed on the fascia. In patients 1, 2, 3, and 6, the FMT was supported with fascia only, which is responsible for the large variability in the retrospective arm of the study. These patients showed worse results in the radiological classification.

**Figure 7:**
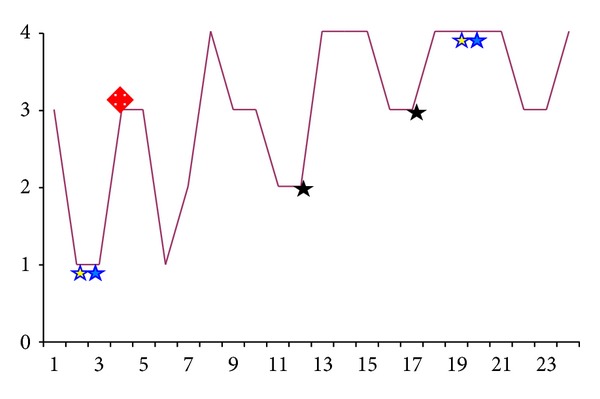
Revision surgeries after radiological evaluation without decoupling. Stars with the same colour indicate revised cases before and after the revision. The red diamond indicates a case which was revised but had no scan available from after the first surgery (retrospective case). AF values in dB.

**Figure 8:**
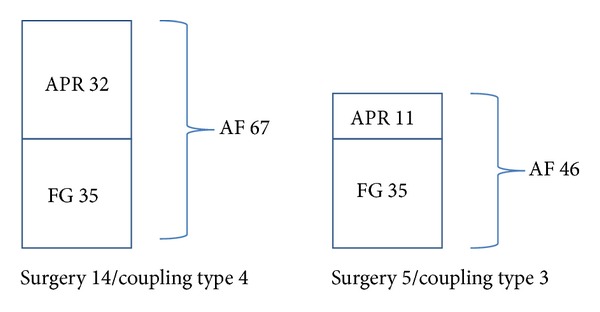
Exemplar estimation of the AF for surgeries 5 and 14 with the specific information attached.

Fascia was used to connect the FMT to the RW in patients 1–7 (the retrospective arm of the study), whereas in patients 8–24 Ivalon was used (prospective arm). Patient 4 experienced a migration of the FMT away from the RW and underwent revision surgery to further stabilize the coupling (the radiograph after revision is depicted in Figure 7). The migration was described 3 months after the first operation, in which the promontory lip was not removed and the FMT was stabilized at the distal end only with fascia. 

The patients were clinically reevaluated after the radiological examination of the FMT position. In some cases, this led to a transtympanic repositioning of the FMT at the RW. An optimized radiological position was achieved in all this patients (Figure 7). All patients are daily users of the VSB system.

## 4. Discussion

Colletti's suggestion to position the FMT at the RW extended the indication range for middle ear implants from pure SNHL to mixed hearing losses.

The validity of positioning the FMT at the RW niche with respect to the clinical outcome is obvious since different groups [6–9] showed a clear relationship between the transfer function and the FMT position at the RW in temporal bone studies. 

However, the surgical challenges linked to the FMT-RW niche coupling account for a high variability in the outcome and should not be underestimated. Preservation of the cochlear integrity is of central importance in this specific approach. Drilling at the ossicular chain or the promontory can lead to a noise exposure of more than 130 db SPL [14, 15] and must be done very carefully [14, 15], for example, lowering the speed of the diamond burr to 1000 rpm. In our series, applying this approach led to no significant postoperative hearing threshold shifts in PTAs. 

Postoperative radiological control is helpful to improve the quality of the surgical approach, to monitor the audiological outcome, and to be able to decide which revisions may be required after cochlear implantation and stapes surgery [10–12]. Although the radiological resolution has improved and increased with the advent of new technologies (e.g., 64-MSCT, flat-panel tomography, and digital volume tomography), it has been shown that there is a difference between temporal bone measurements and radiological estimations. Thus, radiological data can only be applied with limited accuracy to the in vivo situation [16, 17]. Additionally, the spatial, 3D position of the FMT at the RW makes it quite challenging to estimate these distances. In contrast to this, our classification of the FMT position was highly reproducible by independent observers (Fleiss-Kappa score: 0.841). We therefore described 4 different classes of FMT position in the RW niche.

One author suggested monitoring the FMT transfer function at the RW by eBERA or EcoG [18]. In contrast to this, we added the individual residual output of the audio processor to the individual functional gain at maximum preferred volume setting. This so-called audio processor factor (AF) seemed to be closely related to the observed radiological classification (Figure 6). Loudness growth curves could be another option to measure the FMT-RW transfer function. 

**Figure 9:**
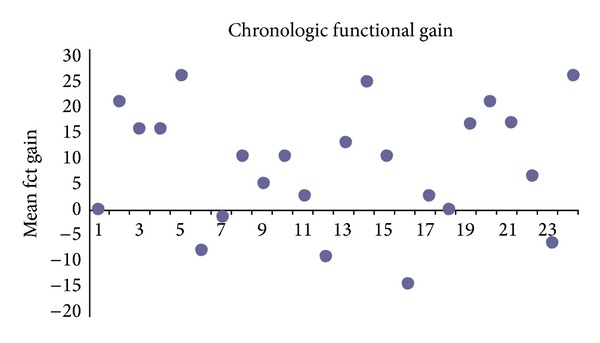
Mean functional gain (overclosure) in dB.

Although not double-checked by eBERA or EcoG, the close correlation between AF and radiological classification led us to assume that it might provide valuable information about the coupling quality of the FMT at the RW. Because of the inhomogeneous audiological indication criteria, in comparison to the classical incus coupling, a correlation between the radiological classification and the pure FG was not observed (Figure 9). Furthermore, the mean FG is worse in RW coupling than the mean FG in classical incus coupling which is related to the differences in audiological indication criteria. 

Additionally, the measured APR cannot be added to the FG to calculate a hypothetical threshold. The individual presence of APR gives information about a relative amount of reserve. The reachable aided threshold is individually variable and influenced by further factors.

The temporal changes of the AF and the radiological classification over time clearly point to the occurrence of a learning curve in terms of improving the FMT-RW coupling. Patients 2 and 3 (retrospective group) emphasize this learning curve. Interestingly, the individual AF of these two patients deteriorated over time after the first AP fitting (Figure 6). In both cases, the promontory lip was not removed, but a closure of the air-bone gap was achieved (as in patients 6 and 12). 

When considering the radiological classification, the necessity of drilling away the promontory lip becomes obvious. This is demonstrated in cases 2, 3, 6, 11, and 12. With or without a partial removal of the promontory lip, only a type 1 or type 2 position was achieved. 

In the rare case of a wide RW niche [19]—as seen in patient 1—a satisfactory AF can also be attained without removal of the PL. 

Another important aspect in achieving optimum coupling is the fixation at the distal end of the FMT in the hypotympanum. In the first 7 cases of our series, only fascia was used for this purpose. In cases 8–24, the fixation was performed with cartilage, which proved to be a stable coupling mode that kept the AF stable as well. 

At the moment, there is no long-term data available in our series to define the influence of fascia, cartilage, and/or their resorption on the AF. The same holds true for the Ivalon placed between the FMT and the RW. While fascia was applied in the first 7 patients, the latter material was used in patients 8–24. It has already been described that these PVA sponges can be invaded by granulomatous tissue [20] also in long-term clinical applications [21]. 

In our revision cases (Figure 7), we were easily able to remove the Ivalon with the surrounding tissue from the RW niche.

The postoperative and retrospective scanning of the FMT led us to revise a number of cases (Figure 7). In all patients, a transtympanic improvement of the FMT position was surgically possible and radiologically proven, with enhancement of the AF occurring as well.

The clinical findings of our series are in line with the temporal bone studies reported elsewhere [6–9].

Comparing the effective radiation dose of a flat panel observation is 1/3 of a temporal bone CT scan [22]. Balancing out the given information against the effective radiation dose makes this observation a reasonable tool. 

Based on our data, a postoperative scan of the FMT position can be strongly recommended for various reasons. This scanning is used as a tool for quality control of the FMT position, and reliably improves the surgeon's learning curve. It should definitely be performed in patients with poor functional gain to help the surgeon make a decision about whether revision surgery may be necessary for positional reasons.
